# Supplementary material for: The nuclear and mitochondrial genomes of amoebophrya sp. ex Karlodinium veneficum
Source: G3 (Bethesda). 2025 Feb 14;15(4):jkaf030. doi: 10.1093/g3journal/jkaf030 (PMC12005148; doi:10.1093/g3journal/jkaf030)
Supplement: jkaf030_Supplementary_Data [file jkaf030_supplementary_data.docx]

**Supplemental Figures**


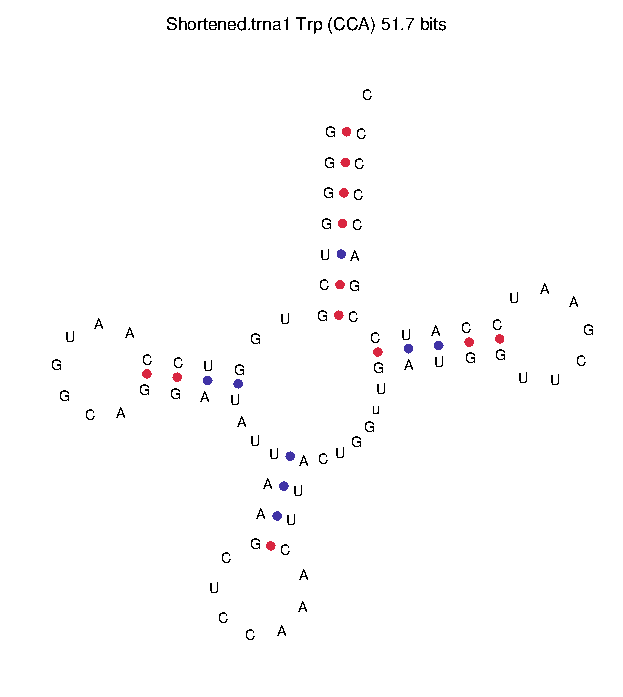


**Supplemental Figure 1.**  The tRNAscan-SE predicted structure for the Trp_CCA_ tRNA with a 4 bp anticodon stem encoded in the *Amoebophrya* sp. ex *Karlodinium veneficum* genome.


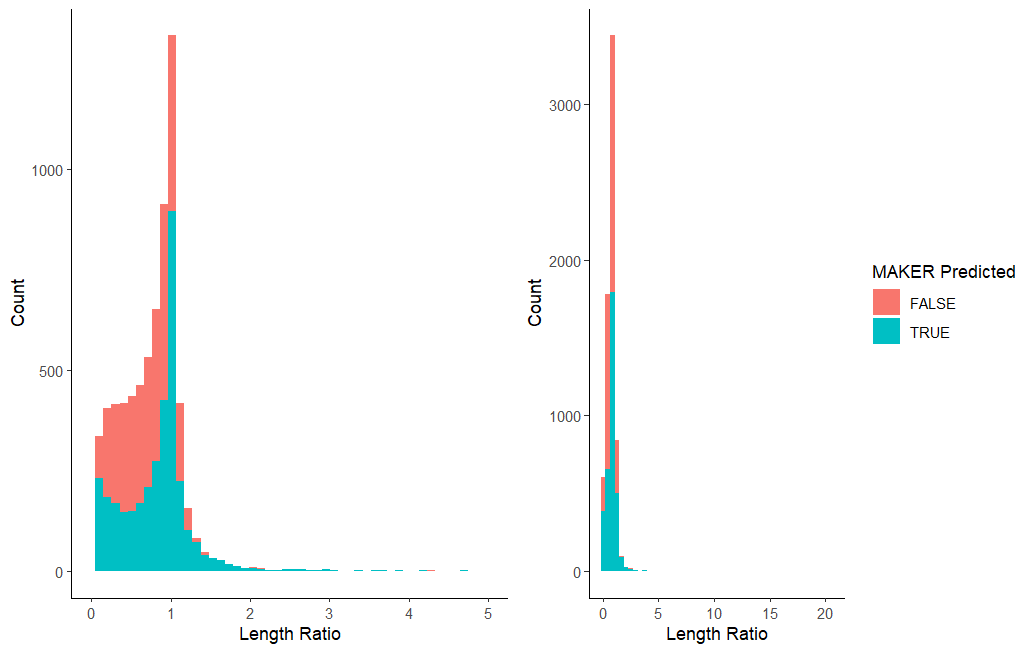


**Supplemental Figure 2.** The distribution of ratios between *Amoebophrya* sp. ex *Karlodinium veneficum* gene lengths and their most similar ortholog in their OrthoFinder assigned orthogroup. The figure on the left shows the distribution constrained to the 0 to 5 range, and the right figure shows the unconstrained distribution. The colors indicate whether the gene was predicted with MAKER or miniprot.


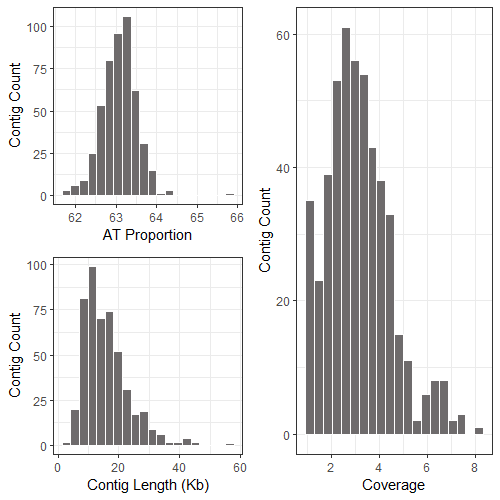


**Supplemental Figure 3**. The distributions of AT proportion, length, and coverage of the putative mitochondrial contigs for *Amoebophrya* sp. ex *Karlodinium veneficum*.


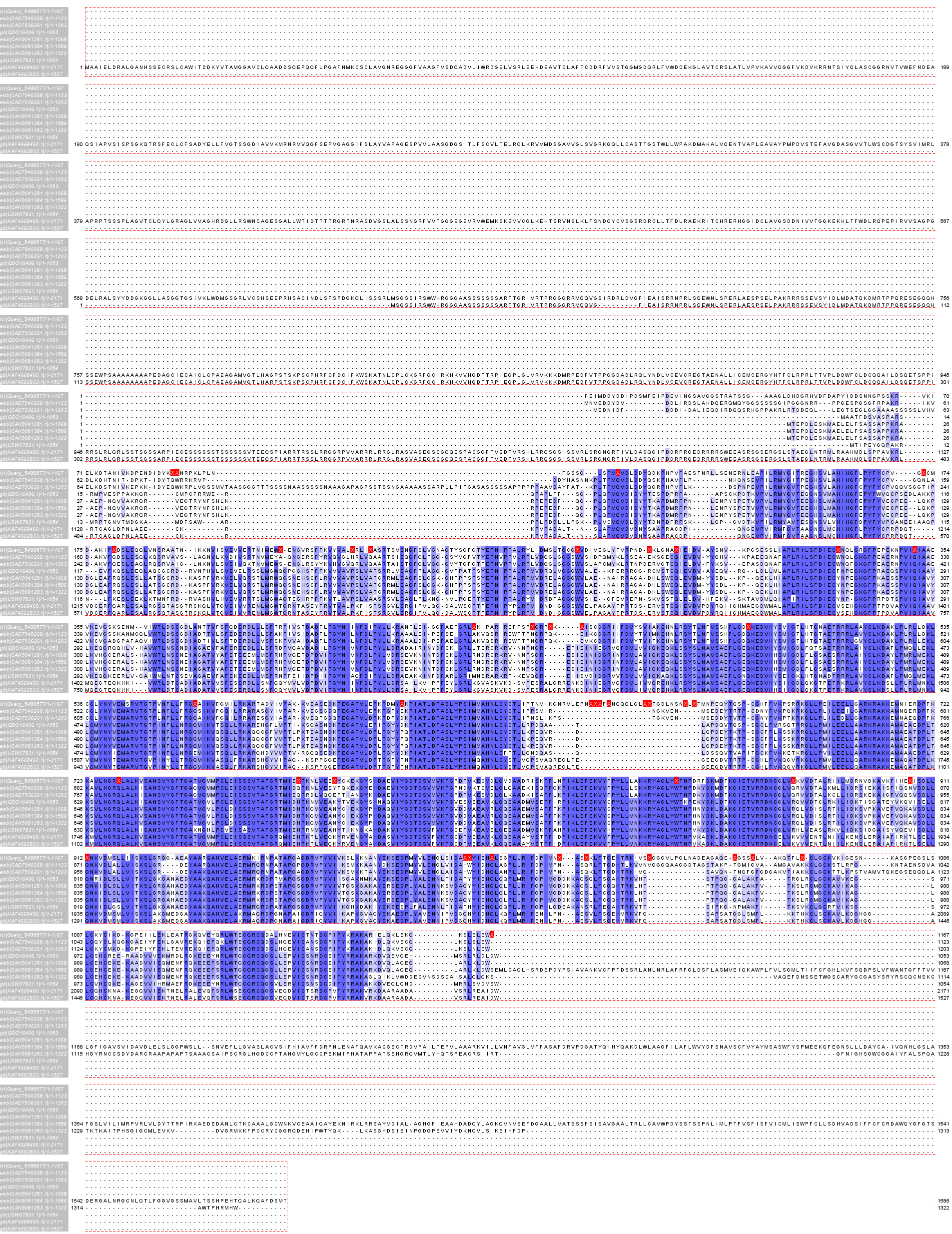


**Supplemental Figure 4**. The alignment of *Amoebophrya* sp. ex *Karlodinium veneficum*’s DNA Polymerase Delta Catalytic Subunit (AmexKv8001) to its top ten BLASTp results in RefSeq’s nr database. Red residues indicate in-frame stop codons in the *Amoebophrya* sequence, and the intensity of blue in the columns indicates the degree of conservation of that column in the multiple sequence alignment.


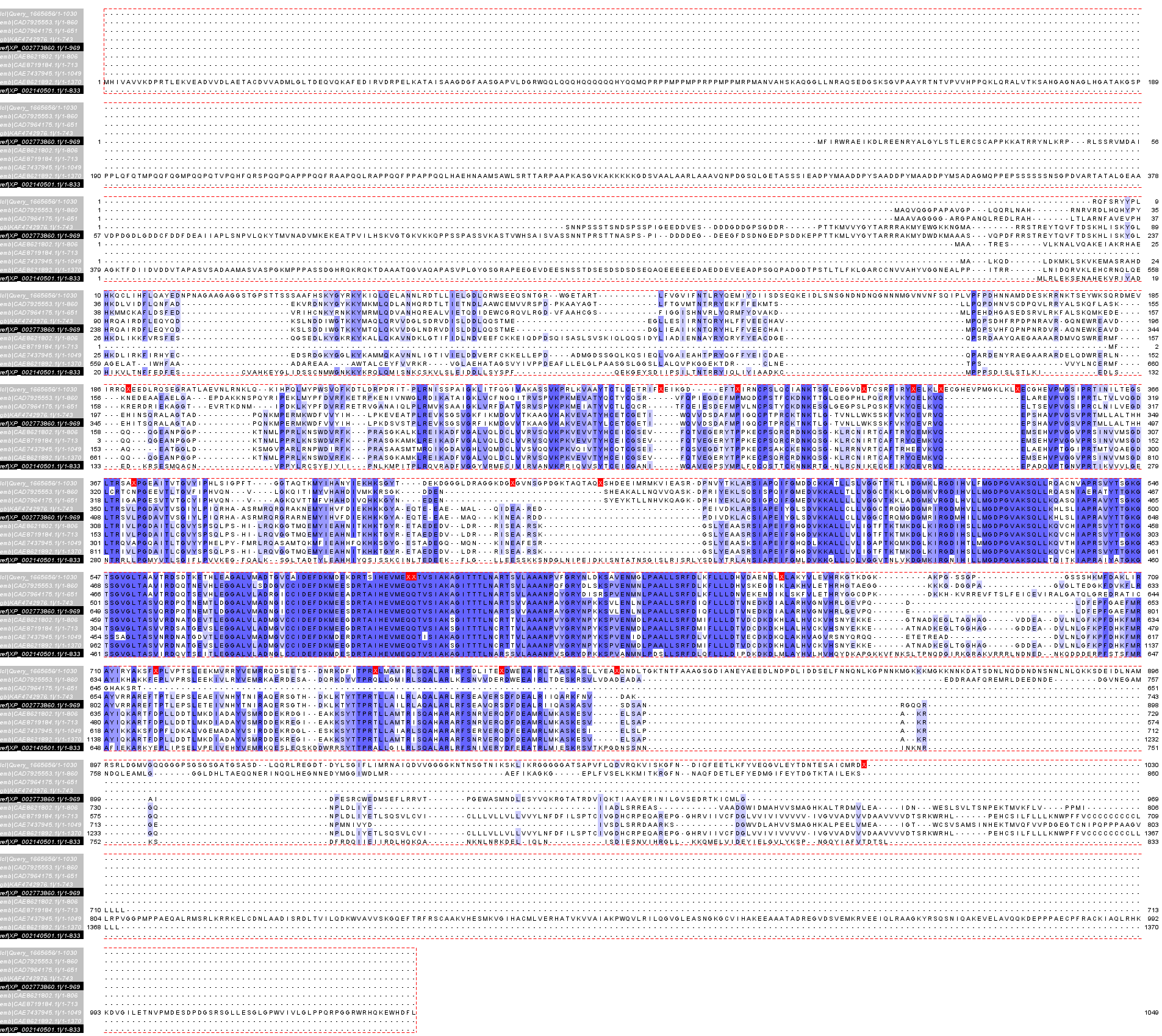


**Supplemental Figure 5**. The alignment of *Amoebophrya* sp. ex *Karlodinium veneficum*’s MCM7 (AmexKv5560) to its top ten BLASTp results in RefSeq’s nr database. Red residues indicate in-frame stop codons in the *Amoebophrya* sequence, and the intensity of blue in the columns indicates the degree of conservation of that column in the multiple sequence alignment.


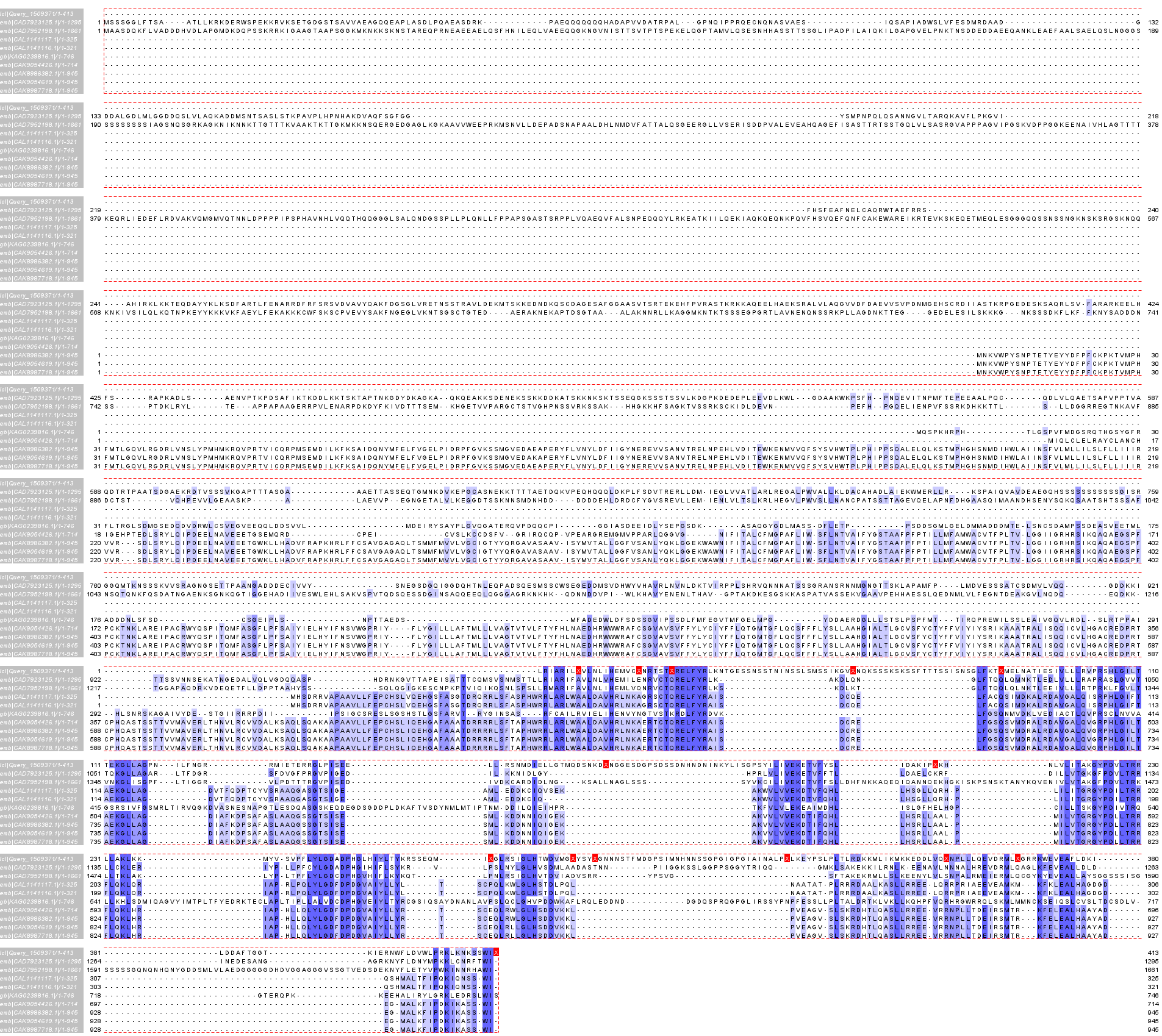


**Supplemental Figure 6**. The alignment of *Amoebophrya* sp. ex *Karlodinium veneficum*’s Spo11 (AmexKv7515) to its top ten BLASTp results in RefSeq’s nr database. Red residues indicate in-frame stop codons in the *Amoebophrya* sequence, and the intensity of blue in the columns indicates the degree of conservation of that column in the multiple sequence alignment.
